# Compassionate Othering: the construction of refugee patients in medical students’ narratives – a qualitative study using *story completion*

**DOI:** 10.1186/s12909-024-05684-9

**Published:** 2024-06-27

**Authors:** Lena Bauer, Andreas Wienke, Amand Führer

**Affiliations:** Institute for Medical Epidemiology, Biometrics, and Informatics (IMEBI), Interdisciplinary Center for Health Sciences, Medical School of the Martin Luther University Halle-Wittenberg, Magdeburger Straße 8, 06112 Halle (Saale), Germany

**Keywords:** Story completion, Medical students, Othering, Refugees, Compassion, Structural vulnerability, Medical education

## Abstract

**Background:**

Refugees remain a marginalized population and are exposed to a variety of discriminatory processes, among them Othering which categorizes people as belonging or not-belonging according to certain ascribed characteristics. We explored how the narrative construction of refugee patients by medical students constitutes a form of Othering.

**Methods:**

Using *story completion*, 124 5th year medical students at the Martin- Luther- University Halle-Wittenberg in October 2019 wrote a fictional story in response to a story stem situated in a medical practice. In a comparative approach, one patient presenting with abdominal pain lacks further characterization (version A) and the other is a refugee (version B). The stories were coded using qualitative content analysis by Mayring with a focus on content and narrative strategies (plot structure and perspective).

**Results:**

We identified four themes: characters, medical condition, access to care and provision of substandard care. The stories were predominantly framed with a medical or an interaction-based plot structure and written from a process-oriented perspective. The themes in version B, supported by their use of narrative strategies, were largely contextualized within the patients’ history of migration. An empathic depiction of patient B and the students’ compassion for the patients facing substandard care were key motifs as well.

**Conclusion:**

The perception of the version B patients predominantly as refugees establishes their construction as an Other. The students’ compassion acts as a representation of societal inequalities and remains an inept response without the tools to counter underlying discriminatory structures. Based on a discourse of deservingness, compassion alone therefore perpetuates Othering and highlights the need for structural competency training in medical school.

## Background

In the last decade, Germany has witnessed a considerable increase in immigration by refugee populations comprised of a wide spectrum of nationalities, ethnicities, and age groups [1]. This development poses a variety of challenges, including numerous for the German health care system. Various structural barriers restrict access to care for patients with a migration background [2, 3]. Furthermore, difficulties in the interaction between patients and medical personnel can also present obstacles to adequate care [4, 5]: Salient factors include an oftentimes inadequate handling of language discrepancies [6, 7], low levels of intercultural competence [8, 9] or medical personnel’s hostile attitude [5, 9–11]. This often results in the exclusion of refugee patients from health care services, in misdiagnoses, and in mistreatments.

From a social science point of view, many of the problems migrants face in the health care system have been linked to Othering [12–15]. Hereby, refugees are subjected to processes that mark them as different from other patients which in turn serves as a justification for their discrimination.

The roots of Othering theory are manifold. The following intends to showcase some of the most relevant strands of theorizing for our topic. We hereby mostly situate ourselves in feminist and postcolonial theory for their usefulness to grasp power dynamics and hierarchies. We do not touch upon other concepts that address alterity or difference [16–18] that are not central to our analysis.

In feminist theory, the concept of Othering dates back to Simone de Beauvoir’s pivotal work *The second sex* [19]. Beauvoir argues that in dominant societal perception man represents the social norm and woman is considered a deviation from said norm. The identity of the woman is therefore constructed in reference to man – more precisely the lack thereof being the defining characteristic. “She […] is the inessential in front of the essential. He is the Subject; he is the Absolute. She is the Other” [19]. Beauvoir not only criticizes the power of definition based in alterity, but she also denounces the resulting legal and economic disadvantages for women.

Another strand of theorizing comes from postcolonial scholarship: In reference to orientalist discourse, Edward Said asserts that “the Orient” has been constructed as the Other in relation to the Subject, “the Occident”, as he is describing the exoticizing and pathologizing portrayal of the Orient prevalent in Europe during the post-Enlightenment period [20]. Said describes a discourse that constructs “the Orient” from a European point of view, in academic contexts and as an imaginative idea in literature. He hereby shows that the contextualized discourse of Orientalism acts as a way of exercising social and political power over peoples perceived to constitute “the Orient”.

Postcolonial feminist scholar Gayati Chakravorty Spivak incorporated these lines of reasoning when she examined the role of marginalized women in postcolonial sites considering current global power dynamics [21–24]. She describes their perpetual marginalization and criticizes the lack of voice of Subaltern women, the Other, in the global feminist discourse. Similar dynamics can be found “along any social dimension” [25]. In recent years, this approach has therefore been taken up and used to describe the situation of various subaltern groups.

In the field of migration studies, an extensive body of literature describes Othering processes pertaining to refugees [12, 14, 25–29]. This line of research is inherently shaped by feminist and postcolonial scholarship and explores the ways in which immigrant groups are designated as subordinate and not belonging. This is oftentimes depicted and criticized as a process of categorization, creating categories of “us” and “them”, such as nationals and refugees [30, 31]. The oversimplifying dichotomy of these categories constructs a social identity and presumed belonging to a social group based on a single characteristic: a person’s residence status. Refugees, as a specific subgroup of immigrants in general, are frequently categorized as one vulnerable Other lacking in agency and individuality and – if proven to be truly in need – deserving help.

Nevertheless, the image of “the refugee” as a national threat persists as well. Olsen et al. argue that the construction of “the refugee” as helpless and vulnerable enables the positioning of oneself as the dominant group and thus serves to ensure the maintenance of existent global power dynamics and national identity [32].

In health care literature, the mechanisms of Othering have been examined, for instance, with regard to public health crises such as the HIV/AIDS epidemic [13, 33–36] and more recently the Covid-19 pandemic [37, 38]. Prevalent themes hereby include the stigmatization of minority groups, the precarity of health care provision as well as differing responses to public health recommendations, creating a mentality of “us-vs-them” [39]. Similar themes have been described in psychiatric and mental health literature [40–44] as well as nursing literature [45].

Another facet of Othering has been highlighted by medical anthropologists studying humanitarian interventions and its moral legitimization as acts of compassion trying to alleviate suffering [46, 47]. The focus on the suffering body has been criticized as it leads to a practice of favoring certain groups and injuries over others depending on their perceived deservingness [48]. Authors have also pointed out the inherent moral hierarchy between humanitarian actors and those they intend to help [49, 50].

In summary, Othering exemplifies a mechanism that is ubiquitous in the construction of people and the ideas by which they are represented. Individuals are thereby categorized as belonging or not-belonging according to distinguishing characteristics. In the case of refugee patients, the failure to address these processes is especially momentous in a context as sensitive as health and leads to deficiencies in medical practice [51, 52].

As prospective medical professionals, students hold a pivotal role in the provision of health care and on the future trajectory of the discourses pertaining to refugee patients. Still, as of now we know very little about medical students’ discursive approach towards patients with a history of refugee migration. Employing the technique of *story completion*, this study therefore intends to explore the construction of refugee patients by German medical students and assess in how far processes of Othering shape their approach.

## Methods

This study employed a cross-sectional design using *story completion* as a means of data collection.

### Story completion as a research method

*Story completion* is a relatively novel qualitative technique. It presents the study participants with the beginning of a story, the *story stem*. This stem acts as a stimulus intended to elicit a reaction in the participants who are then asked to complete the story [53].

Originally, the method is rooted in the projective tests developed to access the unconscious, and reveal psychopathological truths of the patients [54]. The feminist researchers Celia Kitzinger and Deborah Powell first introduced *story completion* to qualitative research discussing essentialist and constructionist approaches in their research while exploring the social discourses surrounding infidelity in partnerships [55].

Contrary to the early projective tests, the stimulus in *story completion* read in a constructionist frame as suggested by Kitzinger and Powell, prompts participants to draw from familiar social discourses in writing their story. The method was further developed by a handful of researchers mostly focusing on the topics of gender, sexuality, and relationships from a feminist perspective [56–62] and increasingly in health research as well [63–66].

Since it asks participants to write fictitious stories and thus shifts focus to a hypothetical scenario, *story completion* is especially suited for topics dealing with sensitive and controversial issues, where the social desirability bias might otherwise be particularly strong [53]. Considering the controversies surrounding refugees in Germany, study participants might be hesitant to disclose their views and attitudes directly, since this debate is often perceived as polarized and emotionally charged. Therefore, *story completion* offers significant potential in this study that is aiming to explore perception, attitude, and bias towards a marginalized patient population.

### Story stems

The *story stems* were developed to present a familiar, concise scenario that could evolve into a myriad of directions. The patient characteristics vary only slightly between the two stems: the patient is either described simply as a young, male patient without any further ascriptions (an “unmarked” patient) or a young, male patient with a history of refugee migration (a patient “marked” as a refugee). Both stems were piloted with a group of 13 medical students that were from another semester than the ones recruited for the study to avoid data contamination [67]. After the pilot, the *story stems* were slightly adjusted, resulting in the following two versions used for the study:

Version A:

*„You are doing a clinical rotation in a primary care practice. On a Monday morning, a 22-year-old patient presents with abdominal pain.”*

Version B:

*„You are doing a clinical rotation in a primary care practice. On a Monday morning, a young patient presents with abdominal pain. He is 22 years old and fled to Germany a year ago.”*

The participants were given instructions to be as creative as desired. They were asked however, to write at least 10 lines or 200 words. We provided them about 20 min to write the story, fill out a questionnaire on empathy, which will be the focus of a follow-up paper, and answer questions on their demographic characteristics.

### Patient A: the norm (?)

The research question as well as the comparative stem design need to be considered within the context of an already established societal framework defining social norms. Even though it would certainly be interesting and necessary, an in-depth discussion of the intricacies of the current scientific debates on questions surrounding the “unmarked norm”, the Subject opposite the Other, is beyond the scope of this article. While we are acutely aware that our comparative study design might reproduce the duality of Othering mechanisms, it allows us to juxtapose both patients, position the unmarked patient A as a point of reference and investigate the narrative construction of our primary group of interest further.

### Sampling and data collection

According to previous research, a sample size of at least 10 participants per *story stem* variation is recommended [53, 68]. To provide ample data, we aimed to recruit all 5th -year medical students enrolled in the compulsory class “Introduction to Social Medicine” who attended the class during the data collection, a total of 166 students. To reach a high response, the students were personally approached by the first author and invited to participate during the class, which is taught by the research team, in October 2019. Students were randomly assigned a version. The survey was carried out in a pen-and-paper format.

### Demographic properties of the sample

Out of the 166 students that were approached by the researchers, 128 students (77%) agreed to participate. Of these, four participants only filled out the empathy questionnaire and declined to write a story. Thus, out of the 124 stories, version A was completed by 53 participants, version B by 71 participants, one of whom did not write a cohesive story but discontinued mid-sentence.

The mean word count was 132 words with a range of 43 to 245 words per story. Almost all participants (*n* = 115) wrote their stories during the time slot given in the seminar, while nine turned their stories in later. Approximately one third of the participants identified as male (*n* = 43), two thirds (*n* = 81) as female and one as gender diverse. Three participants did not reveal their gender. The age range was 21 to 41 years (median = 24 years); most were between the ages of 22 and 26 years (76%).

To distinguish between the study participants and the fictional students in the stories, in the following we use the term “participants” to refer to the actual students that participated in the study while using the term “students” to refer to the fictional students that appear in the stories.

### Qualitative analysis of the story stems

The stories were coded using the software MAXQDA Plus (Version 2020). To prevent bias during the analysis, the stories were anonymized and blinded for the *story stems* beforehand.

We coded the data using the qualitative content analysis approach by Mayring [69]. During the process, we focused on promising ideas and patterns, first exploring the stories’ contents: How are the characters portrayed? Which medical conditions are ascribed to the patients and which corresponding etiologies are suggested? How is access to care described? Hereby, especially the depiction of restrictions to adequate care were of interest: Are there any barriers to adequate access? How do the stories deal with potential barriers? How do these affect the care patients received? Here, we focus on the depiction of refugee patients receiving substandard medical care.

In a second step, we explored two narrative strategies. Analyzing narratives allowed us to examine a story through a more comprehensive lens and helped us understand the reasons and ways in which the stories are constructed [70]. First, we were interested in the underlying structure defining the plot, or rephrased: What framework or idea drives the unfolding of the story? Second, we examined the perspective of the story, in this case referring to which characters or aspects are central to the story. That is, who or what do we learn about the most?

To ensure the reliability of our analyses, we assessed the intercoder reliability following Mayring’s guidelines for a discursive approach [71, 72]. After an introduction to the categories, the supervising researcher coded a random sample that constituted 10% of the data. Subsequently, we discussed the discrepancies in the codes and resolved them, if possible.

### Data interpretation

Following the coding process, we calculated the relative frequencies of the codes using the software MAXQDA Plus. To enable comparisons, descriptive statistics were performed after stratification for the two *story stem* versions, and for participants’ gender.

To illustrate the categories, we present data extracts from the stories below, identifiable by a code as well as by the patient’s (un-)markedness indicating which version the participant received. They have been translated into English and spelling errors have been corrected to aid readability.

## Results

This section will demonstrate the study’s findings according to the main themes of the content analysis: the portrayal of the characters, the depiction of the medical conditions and the access to care as wells as the description of substandard care. In a final section, the narrative strategies will be illustrated.

While most of the stories took place in a real-world setting, two participants displayed their creativity and wrote rather fantastical stories:

*„As I pushed more forcefully, suddenly his abdominal wall ruptured and an alien-like creature jumped out towards me. It had a slimy consistency and smelled like rotten eggs. I tried to capture it, but it was moving too fast and disappeared with a leap through the cracked window of the exam room.” (B27)*.

### Content analysis

- Characters.

In most stories, the two main characters were the patient and the medical student. The following section will highlight how they were portrayed differently in the story versions.

The history of refugee migration mentioned in the stem was crucial to the description of the marked patient in the stories. Some participants referred to him as belonging to the groups of “refugees” (B28) or “displaced persons” (B56). Others emphasized that the patients showed up “like every other urgent patient” (B1) and would therefore be treated like any other patient, irrespective of their insurance status as refugees (B18). Similarly, one participant referred to the patient’s origin while describing his appearance simply as “südländisch” (B35), thus implying a certain generalized look due to his “southern” heritage.

In both versions, the patient was generally depicted as being open-minded, studious, and polite. Some version B stories however, specifically emphasized this and were “really impressed [by] his open and polite nature” (B21). Others stressed the patients’ cooperativeness and compliance: the refugee patient “tries visibly” (B2) to explain his history, yet still failed “despite all of his efforts” (B63).

While the marked refugee patients were depicted sympathetically throughout the stories, a reluctant or difficult attitude, was notably ascribed only to unmarked patients (in 10 out of 53 stories pertaining to him):

*„Since he didn’t have insight into his disease and he only wanted something to counter the pain, it was difficult to convince him of how to proceed.” (A42)*.

In most stories the medical students also played an essential role. They were twice as likely to show positive and friendly attitudes towards the refugee patient than towards the unmarked patient. This was apparent in their desire to help (B4), “to calm him down and relieve his anxiety” (B16) or in complimenting his language skills (B48). Some participants described students that were eager to emphasize their wish to help, explaining that the patient’s well-being was dear to their heart (B22).

None of the stories described a deliberately negative attitude of the students towards the patients.

Summing up, noteworthy differences between the two versions include the emphasis put on the marked patients’ history of refugee migration, the consistently positive characterization of the refugee patient in comparison to the unmarked patient as well as the medical students’ sympathetic and compassionate attitude especially towards the marked patient.

b) Medical condition.

This second main category will take a closer look at the medical condition of the patients in the stories and their underlying etiologies.

It is important to note that the patients did not receive a diagnosis in all stories: In some, this fact was omitted, whereas in others, assigning a diagnosis to the patient played no significant role for the storyline. Interestingly, more than 80% of the patients in the unmarked version A were assigned a diagnosis, compared to roughly 50% of the marked patients in version B. Overall, appendicitis was the most common diagnosis. However, this was not the predominant diagnosis in version A stories. Here, more patients, approximately one-fifth, were suffering due to psychosomatic abdominal pain.

Oftentimes, more complex storylines lead up to these diagnoses and upon examination of the underlying etiologies, with an interesting difference between the two groups. In version A, the etiologies were largely grounded in the patients’ professional lives: All psychosomatic diagnoses, as well as four other diagnoses, were explained by the fact that the unmarked patients were experiencing a stressful situation at their job, school, or university.

For the marked patients however, the etiologies were consistently contextualized within their migration experience. The psychosomatic abdominal pain diagnosed in the six patients in version B was solely grounded in the patients’ status as refugees, the trauma experienced while fleeing their home country and the ensuing difficulties faced in Germany:

*“In addition, he is under severe psychological pressure, because his family still lives in his home country and he is very worried.” (B24)*.

One of the refugee patients was additionally diagnosed with depression “because the memories still haunt him” (B4). Furthermore, one story even painted a picture of repeated abuse of refugees by security guards at the shelter for asylum seekers:

*„Many of his roommates seem to be also exposed to regular physical abuse.” (B20)*.

Finally, other stories suggested quite a different scenario: Several patients were accused of or diagnosed with feigning their condition. The unmarked patients in version A did so hoping to receive a doctor’s note for sick leave from work, school or university. In the version B group, one patient was accused of faking after having difficulties communicating with the doctor:

*“After a very quick physical exam, the doctor can’t find critical evidence. According to the doctor, the history consists of rather vague, sometimes contradicting, statements. The doctor is inclined to dismiss the patient as a ‘faker.’” (B75)*.

Another patient did so supposedly with the intention to affect asylum claims:

*“He doesn’t have any complaints and only wants to avoid work or being deported!” (B1)*.

Overall, the analysis of the medical conditions suggests a particular perception of the patients: the unmarked patients were more likely to be assigned a diagnosis at all, to be diagnosed with psychosomatic abdominal pain and to be accused of feigning. Job and school related issues were the predominant underlying reasons in this group. In contrast, those refugee patients whose stories included a diagnosis or etiology, were predominantly depicted as suffering under migration-related trauma.

c) Access to care.

The main barrier impacting access to care in the stories was communication: More than half of the participants writing stories with refugee patients stated language problems, whereas none in the version A stories described any barrier. While in some stories the language barrier was only stated as such, many stories (41%) elaborated on the matter of communication and provided one specific reason for language barriers, namely the patient’s lack of speaking German:

*„Unfortunately, there’s a big language barrier because the patient doesn’t speak German well yet.” (B10)*.

Two stories were even more explicit: Refugees were specifically blamed for lacking knowledge of the language by the doctor in the stories.

*„It’s always the same! Learn German!“ (B1)*.

However, another two participants also pointed towards other reasons such as the doctors’ inability to speak English, delayed access to German language classes for the patients and their recent arrival to Germany:

*„Even though the family practitioner studied English in school, she hasn’t spoken regularly in a long time. Therefore, she had problems communicating with the young man whose German isn’t all too good yet, because it took a couple of months until he received access to language classes.” (B59)*.

These hurdles in communication between the characters oftentimes shaped the clinical interaction and resulted in insufficient history taking. However, almost 40% of the stories outlined an effort to resolve that barrier by engaging an interpreter, switching to English or using gestures and facial expressions. In one story, the student even distributed a brochure in Arabic (B36). These efforts did not always turn out to be successful though. In others yet, the doctor or the medical personnel refused to try alternative ways in communicating with the patients in the first place:

*„The doctor didn’t try to explain some things in English, but instead quickly brushed aside, everything that wasn’t understood.” (B34)*.

In contrast to the stories highlighting language barriers, eight specifically emphasized the lack thereof and pointed out the patients’ ability to speak German sufficiently. In one story, the student even fondly expressed their surprise about the patient speaking fluently:

*„I was really surprised when he said [he has been in Germany for] only 1 year and [he has] also only been studying German since then – he was already really fluent.” (B50)*.

Overall, stories concerning the refugee patient overwhelmingly engage the problem of language-related problems, one way or another. These and other access barriers were a prevalent theme of interest solely in stories with refugee patients – some resulting in substandard care as shown in the following section.

d) Provision of substandard care.

Almost one-fifth of the stories in the marked version B portrayed a situation with insufficient conditions for the patient in which he received substandard care (such as inadequate history taking, lack of diagnosis or lack of adequate treatment). Most stated an unresolved language barrier as the primary reason, some however also cited a stressful environment and work schedule, general prejudice towards refugees and lack of patience of the medical personnel.

The stories dealt with these discriminatory situations very differently. In five stories, the students addressed the inadequacy of the situation and outlined different narratives concerning the lack of equal access to care. Four of them clearly denounced it:

*„I’m sitting helplessly on the side, trying to help with English/gestures/facial expressions from time to time. At which point however the doctor is signaling nonverbally that my actions are not appreciated. After taking a rather bad history, the doctor examines the patient clinically.” (B34)*.

Three qualified the discriminatory actions in the context of a stressful work environment and well-meaning doctors:

*“Due to the other days during this internship, I don’t believe the doctor is racist, but rather ‘just’ stressed.” (B34)*.

Furthermore, some students reflected on the confusion, helplessness, and internal conflict regarding their own role:

*“Am I naïve because I give everyone a chance anyway? Will I lose my patients later too when there’s not enough time? How are you supposed to solve these problems that start in their heads and are deeply engrained even though someone has ‘just’ stomach pain?” (B1)*.

*„This helplessness and powerlessness is difficult to endure and I am slowly beginning to be able to understand the family practitioner a little bit.” (B75)*.

Especially narratives detailing the provision of substandard care to the patients explored a recurring theme: Faced with discrimination of the patients, the students are oftentimes portrayed as compassionate yet helpless mediators next to the – in some cases dismissive – doctors.

Yet, it is important to note that more than half of participants describing a discriminatory situation did not specifically address it as such nor discussed underlying reasons or resulting consequences in their writing. In contrast, in stories with the unmarked patients, barriers to care or a lack thereof were not mentioned and not considered as something that might impair the clinical interaction.

In conclusion, access to care or lack thereof, its presumed causes and its reception were a major theme in the stories with a marked patient but not a relevant topic in stories narrating care for unmarked patients.

### Narrative strategies

Finally, we shifted the focus of our analysis towards different overarching narrative strategies employed in the stories. This following section will examine first the underlying structure of the plot and secondly the central perspective of the stories. It is important to note that we considered there to be only one central perspective in a story, whereas shifts and breaks in plot structure occurred sometimes and thus more than one plot structure per story is possible.

- Plot structure.

Analyzing the plots of the stories, we found two main approaches, a medical and an interaction-based approach to telling the story. In the medical plot, the stories were structured by the typical steps of a patient consultation that usually include taking a history, a physical examination, diagnosis, and treatment options:

*„After taking a thorough history, […] I moved on to the clinical examination. […] After auscultation and palpation as well as testing appendicitis signs, I suspect ‘appendicitis’. […] We immediately refer Mr. Schmidt to the closest hospital for the surgery.” (A33)*.

More than half of the participants applied such a distinct medical plot to their stories. However, stories in response to the unmarked *story stem* A tended to do so more frequently than those responding to version B (71% vs. 48%).

The second most used plot structure is based on the elaboration of interaction between the characters. This interaction can be constituted by verbal and non-verbal communication unrelated to strict medical history taking as well as actions that allow conclusions to the characters’ relationship. Here, the way the characters played off each other was the main driving force for the plot:

*“And then I saw it again, the wondrous transformation happening to the otherwise very correct, usually somewhat ironically-distanced German doctor. He released an avalanche of French kind remarks onto the patient, asked first of all about the family, the advantages of Cameroonian beer and laughed and joked until the grey examination room disappeared into the background and someone displaced found again some comfort. I was sitting on the side, understood only half and yet learned so much.” (B56)*.

Such an interaction-based plot was more prevalent in the version B stories with a marked patient compared to version A (42% vs. 30%) and largely prompted by a migration-related element (such as experiences during the process of migration or language and cultural differences) in the story (77%).

Noteworthy is also the association between stories in which the patients were given a diagnosis and the predominant plot structure: While the unmarked patients, as mentioned above, are generally more likely than the marked patients to receive a diagnosis, the difference is even more prominent when comparing stories using an interaction-based plot. Here, 94% of unmarked patients receive a diagnosis compared to 53% of the marked patients.

b) Perspective.

As a second narrative strategy, we examined the perspective of the stories. That is, we analyzed which characters are central to the stories.

In almost half of the stories, few if any details on the characters were given, no single character stood out and they all remained relatively neutral and bland. Instead, the stories centered around a sequence of actions; we call this type of perspective process-oriented. There were no substantial differences between the versions (45% A vs. 49% B). However, almost 75% of these stories also followed a distinct medical structure. The following example illustrates this common overlap very well:

*“After consulting with the family practitioner, I’m allowed to first examine the patient myself in a separate room. So, I ask him to enter, introduce myself and take a history concerning his symptoms.[…]. Following the history taking, which hasn’t provided any critical diagnostic clues, I ask him to lie down on the examination table and examine his abdomen. I notice abdominal guarding in the upper middle abdomen. This is where he indicates to have the most pain. I remember that he said, he had eaten little, and I suspect a gall bladder infection.*

*Following the clinical exam, I share my results with the family practitioner. After doing an ultrasound, the sonographic criteria point towards a gall bladder infection.” (A32)*.

Other stories, approximately one-fifth, were centered around the patient as the protagonist (21% A vs 24% B). While the other characters tended to appear only marginally, these stories included vivid details on the patients’ characteristics, experiences, or emotions:

*„She enters the doctor’s office with her head down, yet she is wearing very colorful clothes, many necklaces around her neck, earrings and bracelets. After noticing us, she raises her head and smiles at us.” (A5)*.

In some stories (17% A vs 14% B), the first-person narrators acted as the main protagonists and presented a rather intimate perspective in sharing their thoughts and emotions:

*„Everything I’m hearing from Mr. W., makes me very sad and angry. It’s insane what people can endure without going crazy.*

*I stop for a second and think of my life. How good do I have it!” (A52)*.

Few stories however also focused on a rather unusual “character”: the patients’ medical condition (17% A vs 7% B). In those cases, many clinical details on the patients’ symptoms and medical history were revealed:

*„The patient presents in a reduced overall state, fever of 41°C and very strong abdominal pain. […]The patient’s heart rate is 120/min and his blood pressure is 90/60. I examine him clinically and notice abdominal guarding as well as very strong generalized abdominal tenderness.” (A14)*.

Overall, the influence of the two *story stem* versions was most salient in the comparison of plot structures. The use of a medical plot was predominant in the version A stories with an unmarked patient, focusing the narrative on a rather straightforward exchange of medical information. The interaction-based plot, on the other hand, which is more common with version B stories, shifted the narrative towards an exchange mostly prompted by the patients’ refugee status and strongly emphasizing the characters’ relationships. Considering the perspectives, the stories frequently featured a clear sequence of actions with the differences between the two versions remaining rather modest. Yet it is noteworthy that the use of this process-oriented perspective coincided most with a medical plot.

### Summary of results

In conclusion, these results show the key role the patients’ refugee status seemed to play in the construction of the stories. The medical condition, access to care as well as the resulting substandard care are largely contextualized within the patients’ history of migration. In contrast, the stories relating to the unmarked patient do not touch upon such context factors and tend to focus strictly on biomedical topics. Furthermore, the patient’s refugee status prompted an especially empathic depiction of the patients. However, not only the content of the stories is centered on the patients’ history of migration, the manner in which the stories are constructed differs considerably. The empathic depiction is driven by the prevalent use of an interaction-based plot which is rare in the stories relating to non-migrant patients. Providing a fitting framework for the development of the stories, the use of narrative strategies highlights the one-dimensional lens where patients’ migration experience overshadows other (potential) patient characteristics.

In the discussion, we will now delve further into the context of these results and embed them into the theory of Othering.

## Discussion

When applying a constructionist framework to *story completion* – as suggested by Kitzinger and Powell – it allows us to draw conclusions about “contemporary discourses upon which subjects draw in making sense of experience” [55]. Therefore, our data speaks to the construction of refugee patients in the discourses to which the participants are exposed. It sheds light on how refugee patients are talked and thought about in this particular space. In the following section, this paper will therefore discuss through a postcolonial feminist lens how the construction of “the refugee patient” in this study constitutes a form of Othering.

### Othering: perception through a narrow lens

Building on the theoretical background outlined in the introduction, we would like to argue that overall, in the analyzed stories, the patients in version A represent the default, the unmarked norm, and patients in version B the Other. The one factor defining the marked patient is his status as refugee.

Whereas he is also characterized as being male and young, his migration status is predominant in determining his characters’ description as well as his interactions with other characters. While this focus might certainly be partly due to the reproduction of the marker “refugee” in the story stem, it impacts not only the content of the stories but also shapes the narrative structure in a remarkable way.

Being categorized as a refugee overshadows all other possible markers of identity: Neither gender nor age are utilized in such an instrumental fashion. The relevance and key role that is attributed to the patient’s Otherness, his belonging to a specific group – refugees – sets him apart from the unmarked patient. This narrow lens of perception acts as a backdrop for the following considerations on the intricacies of Othering mechanisms in the stories.

Hereby, it is noteworthy that the one-dimensional perspective on the patient marked as “refugee” elicited mostly compassionate, empathic, and more humane approaches towards this patient. While the unmarked patients in the stories were cared for according to the textbook, engagement with the refugee patients was more individualistic, and less structured by medical reasoning. While at first glance, this seems like a favorable approach towards the delivery of health care, it also entails problems, to which we turn in the sext section.

### Othering through compassion

The students’ compassion and explicit concern for the patient is probably the most defining characteristic of stories answering to story stem B. In the following, we want to outline why this raises important questions and signals a deficit of medical education. Hereby, we draw on the debate around the concept of “othering through compassion”.

Othering through compassion describes putative benevolent attitudes towards a group of people, whose social position is typically lower (or marginalized in other ways) compared to the position of the person performing the Othering.

The idea of compassion in general is defined by Didier Fassin as “sympathy felt for the misfortune of one’s neighbor [that] generates the moral indignation that can prompt action to end it” [49]. The students’ compassion for the refugee patient (but not the unmarked patient) as well as their will to act accordingly is evident in the stories, yet it must be considered within the context of an unequal society. While the moral sentiment itself is inherently one of solidarity and acts of compassion naturally intend to strive for equality, they are overwhelmingly only directed towards the vulnerable and destitute, the less powerful.

In this context, the students’ benevolent attitude towards the patient can therefore be seen as an expression of hierarchy. Their felt obligation to engage the patient “as refugee” is innately rooted in asymmetrical societal power relations. Fassin refers to this as “politics of compassion” [49]. Moreover, the symbolic power and identity building attached to the generosity of providing care to vulnerable humans is another aspect worth highlighting in this context: It entails the construction of oneself as a charitable Subject caring for a vulnerable but deserving Other [32].

In addition to the inequalities inherently implicated in the concept of compassion, it does not necessarily result in constructive action resolving the observed conflict. The recognition of a double standard in the provision of care leaves the students in the story feeling helpless and powerless since their attempts to overcome these difficulties in every-day clinical practice mostly remained futile. In the face of structural inequities and systematic barriers to care, they seem to withdraw to a position of sympathy and compassion.

Compassion alone, however, appears to distract from the recognition of the structural political, social, and economic factors affecting the patients’ care. Compassion thus remains a reductionist and inept response without the tools for a constructive examination of the underlying discriminatory structures. Suggesting an insufficient preparation in recognizing and dealing with these structural determinants of health during their medical education, the students in the stories were not able to translate their compassion into the effective tools needed. A reflection of societal inequality, the sentiment of compassion simultaneously perpetuates discriminatory and unequal situations by ignoring their structural underpinnings.

Since the stories take place in a fictional realm, our analysis does not allow for conclusions about the actual perceptions and actions of the study participants. Yet, they point towards a discourse in which refugee patients continue being subjected to a double standard in care while being compassionately treated like a marginalized Other. The largely uncritical reproduction of this discourse in the stories, however, raises the need for a critical examination of this discursive patterns as well as the sensitization of health care providers.

While here the stories do not speak to compassion as a fruitful foundation for change, when dealt with accordingly, it may serve still as a starting point to constructive change. Compassion may spark a conversation about Othering and action to address these health inequalities.

### Methodological reflections and limitations

The *story completion* method has been a fruitful and insightful method for this study. However, it is crucial to acknowledge that it aims to examine discursive phenomena rather than actual perceptions and actions of the study participants. Thus, our finding of “compassionate othering” clearly speaks to the nature of the discourses medical students are exposed to and draw from when writing their stories, but it does not necessarily reflect how participants would act if they were in the position of the student in the story.

Furthermore, while the socially value-laden and structurally important category of “migration” has prompted ample data, the lack of a designated category for patient A may have prompted the coherent use of a standard medical narrative in this version. Marking the second patient with an uncontroversial attribute, such as a hobby, might have provided a more diverse set of data. Still, *story completion* holds a lot of potential and this study aims to contribute to its further methodological advancement.

## Conclusion

The discourse that shaped the respondents’ stories takes place in a system that still systematically lacks attention to the structures producing health inequities. With much of medical education focused on a biomedical paradigm, students and medical practitioners alike fall back on every-day discourses and their gut feeling when confronted with patients that (seem to) require a more biopsychosocially informed approach [73]. Compassion then is not translated into action that challenges the forces creating structural vulnerability for certain types of patients but materializes in the form of intrapsychic conflict.

Yet, universities are in a pivotal position to start adequately preparing future medical professionals to care for the structural vulnerability of marginalized patients [74–76]. In order to be able to provide the best care possible, students need to be trained to recognize the various factors contributing to inequities and be equipped with tools to navigate care for their patients [77, 78]. Hereby, a critical reflection of one’s own role within the overarching power dynamics should be an essential first step to reflect on the question why certain patient characteristics engender compassion (and others don’t). As our analysis highlighted, the discourse shaping our respondents’ stories is still strongly influenced by Othering mechanisms in the perception of refugee patients and therefore stresses the importance of structural competency training during medical education.

## Data Availability

The datasets used and/or analysed during the current study are available from the corresponding author on reasonable request.
